# Learning and distraction: Evidence for cognitive load interference in medical education

**DOI:** 10.1111/medu.70136

**Published:** 2025-12-18

**Authors:** Andrea Storck, Clemens Grahl Römer, Steffen Ansorge, Eva Schönefeld, Michelle Bellstedt, Birte Barbian, Martin Janssen, Konstantin E. Seifert, Dogus Darici

**Affiliations:** ^1^ Department of Urology University Clinics Münster Münster Germany; ^2^ University of Münster Münster Germany; ^3^ Berlin University of Applied Sciences and Technology Berlin Germany; ^4^ Institute of Education and Student Affairs University of Münster Germany; ^5^ Institute of Anatomy and Neurobiology Münster Germany

## Abstract

**Background:**

Distraction may increase cognitive load. Cues may decrease it. But what happens if we cue in distracted learning environments? Does effective instruction buffer against the detrimental effects of distraction?

**Methods:**

In a 2 × 2 factorial experiment, 117 s–year medical students without prior knowledge watched a standardised instructional video on abdominal ultrasound. Distraction was induced via a concurrent mental arithmetic task, and supportive cues were instructed via eye movement modelling examples of an expert's gaze. Image interpretation performance and cognitive load were measured before and after training.

**Results:**

As expected, cueing alone reduced extraneous cognitive load and improved learning. Distraction alone impaired learning. However, when both interventions were combined, the performance benefits of cueing disappeared. Distracted learners receiving cues performed no better than uncued distracted learners, indicating no compensatory effect. Thus, distraction not only weakened learning but blocked the effectiveness of instructional benefits.

**Conclusions:**

The disappearance of instructional benefits under distraction suggests a *load interference mechanism*: Learners cannot benefit from helpful educational instructions when their working memory is already taxed by competing demands. Importantly, this blocking effect represents more than a simple additive effect—it demonstrates a qualitative breakdown where helpful instructional elements become ineffective rather than merely weakened. We discuss the implications for medical education in increasingly distraction‐rich learning environments characterised by AI, smartphone notifications and electronic health record alerts.

## Abstract 2

A study of 117 med students shows that distraction erased gains from gaze cueing in ultrasound training showing evidence of cognitive load interference. In distraction‐rich environments, protecting attention may matter more than optimizing teaching. #MedEd

## 1 INTRODUCTION

In a hyper‐connected world, medical students are increasingly exposed to task‐irrelevant information, from technical notifications and multitasking demands to pervasive screen use. These distractions have been linked to declines in sustained attention, working memory (WM) and learning efficiency,^1^ posing a growing challenge to instructional effectiveness in both classroom and clinical settings. Reflecting these concerns, there is a growing debate in higher education about banning electronic devices in lectures and practical courses, with some institutions implementing strict device policies in an attempt to preserve students' cognitive focus and learning outcomes.^2^

The goal of the current study was to examine how a well‐established educational intervention–cueing–performs under distraction. Cueing refers to techniques that direct learners' attention to task‐relevant information.^3^, ^20^ As such, it holds the potential to actively restore or refocus attentional resources in cognitively demanding situations. To investigate this interaction, we employed a factorial design that allowed us to examine both interventions independently and in combination.

### 1.1 Theoretical background

The current study builds on *Cognitive Load Theory* (CLT), which provides a framework for understanding *how* distractions might affect learning.^4^ According to CLT, learning is limited by the capacity of the WM, which serves as the bottleneck for information processing. Current models distinguish two main types of cognitive load: intrinsic cognitive load (ICL), determined by the inherent complexity of the learning material; and extraneous cognitive load (ECL), imposed by the instructional presentation method or unrelated activities. Cognitive load can be influenced by the instructional design of the learning materials aiming to reduce ECL and hence improving the efficiency of the learning process.

One such strategy is eye movement modelling examples (EMME), a sort of gaze cueing^3^ that have been successfully used in instructional videos, for example, by superimposing expert gaze pattern onto a training video (e.g. ^5^, ^6^). By directing learners' attention to essential elements through demonstrated eye movement patterns, EMME may help learners to select relevant from irrelevant information, thereby lowering their ECL. This approach leverages selective attention mechanisms to ‘filter out’ distracting information ^7^ and facilitate the integration with long‐term memory.

In contrast, traditional CLT‐theory discourages distractions or *seductive details*, which impose additional extraneous load unrelated to the primary learning objective.^8^, ^9^, ^10^, ^11^ For example, when learners engage in distraction, fewer cognitive resources remain available for following the relevant information, resulting in slower response times and learning loss.^12^ Similarly, engaging in a concurrent task (‘multitasking’) requires sustained executive control to hold intermediate results in WM and inhibit interference from competing task demands. This process requires executive attention to maintain the task goal and control attention in a top‐down fashion. Thus, distractors might pose a higher strain on this limited resource. As such, distractions consume limited‐capacity resources and overload the central executive and phonological or visuospatial subsystems that are essential for processing and integrating new instructional material.^13^

### 1.2 The present study

In the context of increasingly distraction‐rich medical education environments, we asked if a well‐established instructional design (i.e. EMME) maintains effectiveness when students face concurrent cognitive demands. According to CLT, EMME and distraction would lead to opposite effects on ECL. Specifically, EMME is designed to reduce ECL,^6^ while distraction might increase ECL. From a CLT perspective, the critical question becomes whether the load‐reducing benefits of EMME can compensate for the load‐increasing effects of distraction, or whether distraction overwhelms the instructional support that EMME provides.

To simulate this condition, we applied a dual‐task paradigm,^14^ pairing sonography training with or without EMME cueing (primary task) with a concurrent distraction task (secondary task). This design allowed us to test whether instructional cueing maintains its effectiveness when learners face the divided attention characteristic of distraction‐rich educational settings.

We anticipate three possible outcomes:

- Low interference: Cueing remains equally effective despite distraction, such that distracted learners receiving cues show an equidistant increase in performance as undistracted learners with cues. Here, EMME's attentional guidance enables the central executive to maintain selective attention on relevant content, sufficiently reducing extraneous load to offset distraction. This effect is additive but compensatory: Cueing reduces ECL by roughly the same amount that distraction increases it, resulting in preserved performance.
- High interference: Distraction eliminates the benefits of cueing, leaving distracted learners with cues performing no better than those without cues. This indicates that extraneous load from distraction overwhelms the central executive's capacity for selective attention, depleting WM regardless of cueing. Here, cueing and distraction could interact nonlinearly, such that once total load exceeds a critical threshold, additional guidance cannot restore performance and a saturation or overload effect occurs.
- Partial interference: Cueing mitigates (but does not fully counteract) the detrimental effects of distraction, resulting in performance that lies between distracted learners without cues and undistracted learners with cues. This reflects incomplete load reduction where the central executive manages some but not all competing attentional demands.

## 2 METHODS

### 2.1 Study design

This randomised controlled trial employed a factorial pre‐post‐design. The study was reviewed by the ethics committee of the university (‘Ethik Kommission Westfalen‐Lippe’) and deemed not to require formal medical ethics approval (reference: 2023‐631‐f‐N). All procedures were carried out in accordance with the Declaration of Helsinki and its later amendments.

### 2.2 Participants

A priori power analysis (G*Power; Faul et al., 2007) with medium effect (*f* = 0.25), alpha = 0.05, power = 0.80, *r* (pre, post) = 0.50 indicated a minimum of *N* = 68 participants. Second‐year medical students from the University of Münster were recruited prior to a mandatory course for anatomy and imaging. Participation in the study was voluntary, and informed consent was received. Inclusion criteria were (1) completion of regular anatomical curriculum, (2) no prior knowledge in medical imaging or sonography and (3) voluntary participation with informed consent. One participant with extreme values (>3 SD) was excluded after data inspection.

### 2.3 Procedure

The study was conducted in a controlled multimedia laboratory equipped with standardised computers and noise‐cancelling headphones. Participants completed the study individually using an offline HTML5‐based study environment to ensure consistent conditions and prevent external influences. Students remained blinded to their intervention conditions throughout the study duration.

**FIGURE 1:**
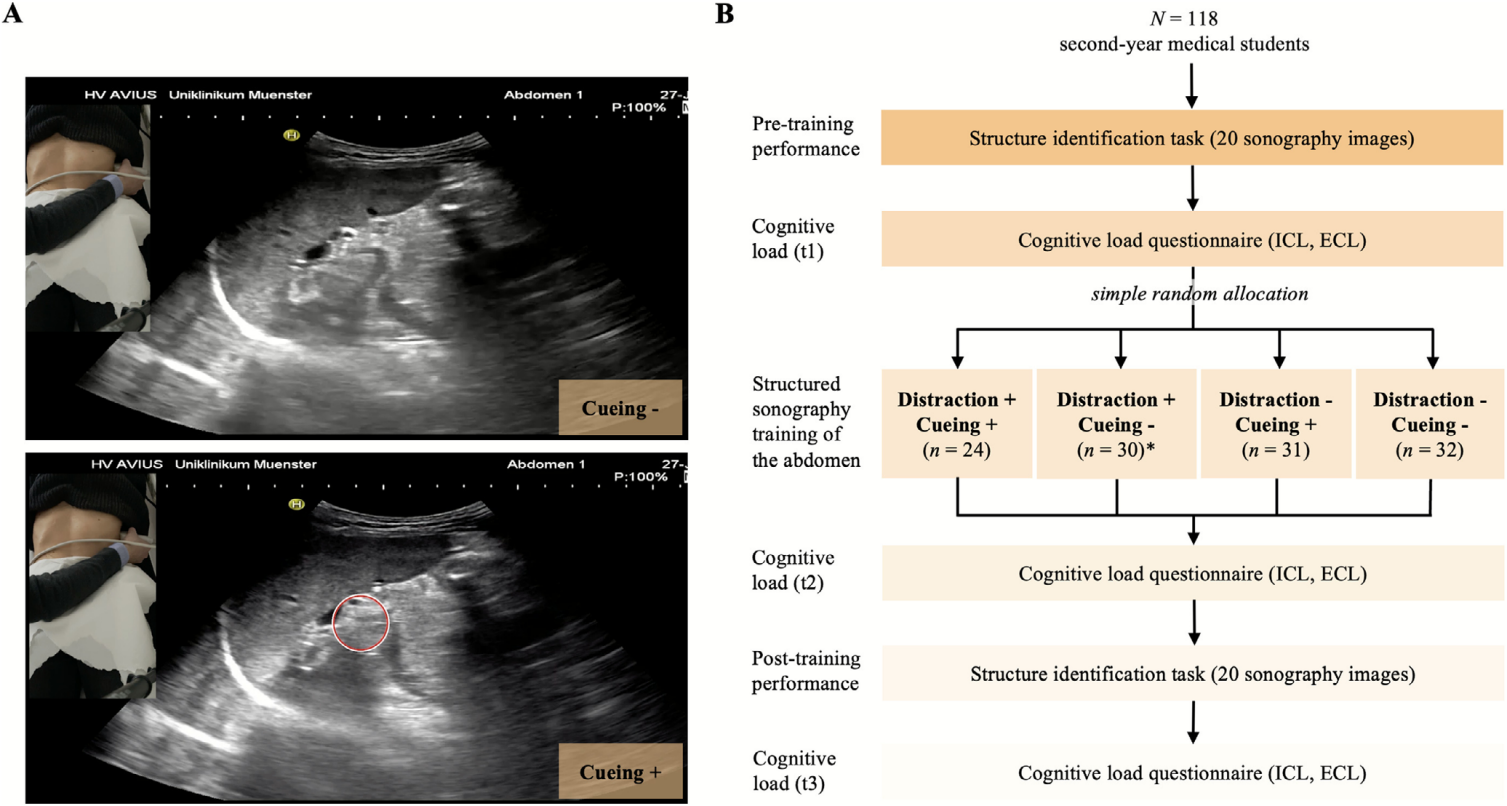
Experimental conditions and study design. (a) Example still frames from the abdominal sonography training video showing the two cueing conditions. In the **Cueing −** condition (top), learners viewed the unmodified sonographic video. In the **Cueing +** condition (bottom), gaze‐based visual cues were overlaid as a semi‐transparent moving circle (red) indicating the expert's point of visual focus in real time. (b) Flowchart of the study procedure. Note that ICL and ECL was measured directly after the pre‐test (t1), after the training (t2), and after the post‐test (t3). Specifically, t1 and t3 ratings referred to the image interpretation task, while t2 ratings referred to the training phase.* Numbers do not add up due to participant exclusion in this group. ICL = Intrinsic Cognitive Load; ECL = Extraneous Cognitive Load. [Color figure can be viewed at wileyonlinelibrary.com]

The study followed a structured timeline as illustrated in Figure 1:

1. Pre‐intervention phase (15 min): Participants completed sociodemographic questionnaires, followed by a pre‐test of sonographic image interpretation and initial cognitive load assessment.
2. Randomisation: The platform automatically allocated students to one of four experimental groups using simple random allocation based on their assigned experimental condition
3. Training intervention (5 min): Participants viewed an audio‐commented routine sonography examination of the abdomen performed by a senior radiologist with 10+ years of experience. Technical details in the Supporting Information supplements. The sonographic video included pictograms indicating probe location and orientation.
4. Post‐intervention phase (20 min): Participants completed post‐training cognitive load questionnaires, repeated the sonographic image interpretation test (identical items in randomised order) and provided final cognitive load measurements.
5. At the last page of the intervention, participants were asked if they had fully engaged in the study.

### 2.4 Visual cueing

Visual cueing was implemented through EMMEs as described in Darici et al.^5^ Technical details are in the appendix. Previous research demonstrated that this intervention significantly improved participants' image interpretation performance by directing attention to task‐relevant areas.

### 2.5 Distraction

Cognitive distraction was experimentally induced using a dual‐task paradigm following Madsen et al.^12^ and Sieg et al.^10^ Participants in the distraction condition were instructed to perform continuous mental subtraction (serial sevens: 400–7 = 393, 393–7 = 386, etc.) while viewing the sonographic video. This arithmetic task creates substantial cognitive load and competes for WM resources that simulates conditions where health care professionals must process multiple information streams simultaneously. When doing mental arithmetic, the central executive decides what to attend to (the numbers), suppresses distraction and coordinates the phonological loop and visuospatial sketchpad. While our mental subtraction paradigm may appear artificial compared to naturalistic clinical distractions, it specifically targets the same WM subsystems that are compromised during internal distractions, making it a proxy for understanding load interference principles (see Hitch et al.^33^).

### 2.6 Measurements

Measurements included sociodemographic variables of age (free‐text response) and gender (man/woman/non‐binary), along with 20 single‐choice items (1 of 5) related to image interpretation performance in sonography (before training: Cronbach's *α* = 0.474, after training: *α* = 0.345; *r* = 0.53, 95% *CI* = 0.39;0.65). We measured prior knowledge in anatomy with 10 single‐choice items (1 of 5; *α* = 0.471). Cognitive load was measured using the scale by Klepsch et al.^15^ with two items for ICL (t0: *α* = 0.633, t1: *α* = 0.589, t2: *α* = 0.741) and three items for ECL (t0: *α* = 0.744, t1: *α* = 0.569, t2: *α* = 0.785) respectively.^16^ Preparatory engagement and anatomical knowledge were assessed as covariates. Secondary‐task performance was operationalised as the lowest number of students reached in the serial‐seven task.

### 2.7 Statistical analysis

Statistical analyses and visualisations were performed using R.^17^ Code in the Supporting Information. An ANOVA for repeated measures was conducted with time (pre‐training versus post‐training) as within‐subject, the two factors gaze Cueing (+/−) and Distraction (+/−) as between‐subject, and image interpretation performance score as dependent. Secondary analyses examined cognitive load change across conditions and time points using similar mixed‐design ANOVA. Effect sizes were reported as partial eta‐squared (*η*_*p*_^2^), interpreted according to Cohen's conventions: *η*_*p*_^2^ = 0.01 (small effects), *η*_*p*_^2^ = 0.06 (medium effects) and *η*_*p*_^2^ = 0.14 (large effects).

## 3 RESULTS

### 3.1 Participants

The final sample (*N* = 117) representing around 80% of the semester cohort was evenly distributed across the four experimental groups. Self‐reported ultrasound‐related pre‐knowledge scores were low and showed moderate variability. Preparatory engagement and anatomical knowledge scores were also generally balanced across groups, with no substantial group differences evident prior to the intervention.

### 3.2 Cueing benefits learning only when attentional resources are available

Post‐test performance was examined using a mixed ANOVA. The analysis revealed a main effect of time, *F*(1, 224) = 9.23, *p* = 0.003, *ηp* ^2^ = 0.040. The main effects of cueing, *F*(1,224) = 0.18, *p* = 0.674, *ηp* ^2^ = 0.001 and distraction, *F*(1, 224) = 0.68, *p* = 0.411, *ηp* ^2^ = 0.003, were not significant. There were significant interactions of time × cueing, *F*(1, 224) = 5.07, *p* = 0.025, *ηp* ^2^ = 0.022, time × distraction, *F*(1, 224) = 7.17, *p* = 0.008, *ηp* ^2^ = 0.031 and cueing × distraction, *F*(1, 224) = 6.46, *p* = 0.012, *ηp* ^2^ = 0.028. The three‐way interaction did not reach significance, *F*(1, 224) = 1.99, *p* = 0.160, *ηp* ^2^ = 0.009.

A sensitivity analysis using reliability‐corrected gain scores confirmed a main effect of cueing, *F*(1,113) = 16.45, *p* < 0.001, *ηp* ^2^ = 0.11, 95% *CI* [0.03, 1.00]; *ηp* ^2^ = 0.095, 95% *CI* [0.026, 1.00] and a cueing × distraction interaction, *F*(1,113) = 5.11, *p* = 0.026, *ηp* ^2^ = 0.04, 95% *CI* [0.003, 1.00]; *ηp* ^2^ = 0.034, 95% *CI* [0.0001, 1.00], while distraction alone remained non‐significant (*p* = 0.108). Secondary‐task performance was moderate overall (*M* = 104.89, *SD* = 111.83), and it was not significantly associated with post‐test diagnostic accuracy, *r*(53) = −0.12, *p* = 0.391, 95% *CI* [−0.37, 0.15].

**FIGURE 2:**
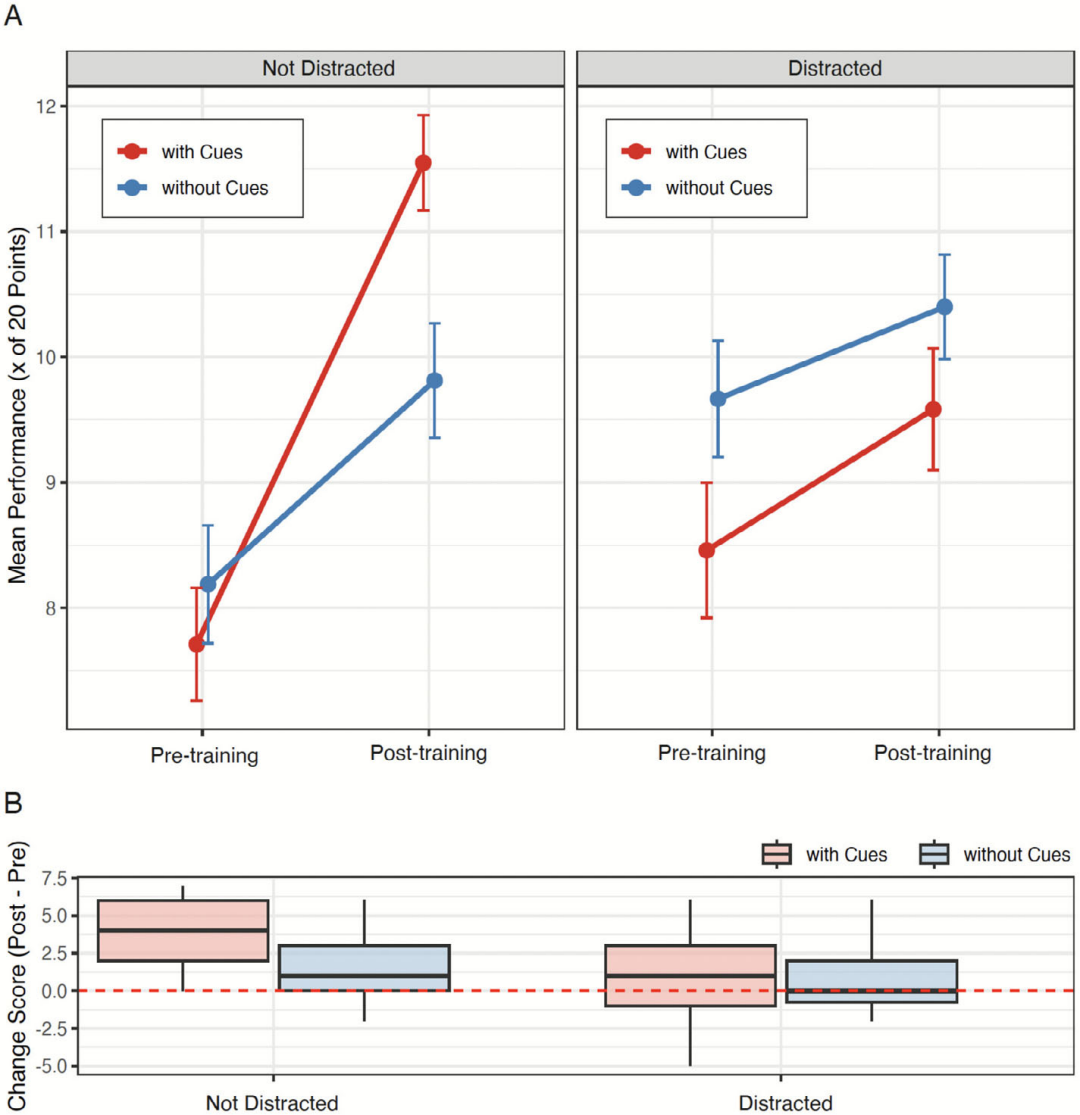
Cueing benefits learning only when attentional resources are fully available. (a) *Mean* pre‐ and post‐training performance (maximum 20 points) in the structure identification task, separated by distraction (columns) and cueing condition (colours). Error bars indicate ±1 *standard error*. Without distraction, cueing produced substantial learning gains, whereas under distraction, cueing benefits disappeared.(b) Distribution of change scores (post‐test minus pre‐test performance) for each condition. The largest gains occurred in the no‐distraction + cueing group, while all other groups showed smaller or negligible improvements. The dashed red line marks zero change. [Color figure can be viewed at wileyonlinelibrary.com]

As shown in Figure 2A, when learners were undistracted, cueing produced substantial learning gains, with mean scores rising from *mean* 7.7 to 11.5 out of 20. In contrast, undistracted learners without cues improved only from 8.2 to 9.8, demonstrating a significant benefit of cueing when attentional resources were fully available.

However, this benefit compromised under distraction. Distracted learners receiving gaze cues improved marginally (8.5 to 9.6), performing almost identically to their distracted, no‐cue counterparts (9.7 to 10.4). The near‐parallel performance trajectories in the distracted groups (Figure 2A, right) highlight the failure of cueing to enhance learning when cognitive capacity was taxed.

An ANOVA change‐score analysis (Figure 2B) corroborates the effect of experimental condition, *F*(3, 113) = 12.37, *p* < 0.001, *η* ^2^ = 0.250, 95%CI [0.15; 1.00]. Tukey‐adjusted post hoc comparisons showed that participants in the cueing + no‐distraction group achieved greater performance gains than the cueing + distraction (*M*_*diff*_ = −2.71, *SE* = 0.59, *t*(113) = −4.61, *p* < 0.001, 95%*CI* [−4.25; −1.18]), no‐cueing + distraction (*M*_*diff*_ = −3.11, *SE* = 0.56, *t*(113) = −5.60, *p* < 0.001, 95%*CI* [−4.55; −1.66]) and no‐cueing + no‐distraction (*M*_*diff*_ = 2.21, *SE* = 0.55, *t*(113) = 4.06, *p* < 0.001, 95%*CI* [0.79; 3.64]) groups. All other comparisons were nonsignificant (*p*s > 0.37). The informative hypotheses approach also supports the high interference hypothesis (*Bayes Factor* = 8.698, *Posterior Model Propability* = 0.897), details in the appendix.

### 3.3 Cueing reduces cognitive load during training, particularly under distraction

**FIGURE 3:**
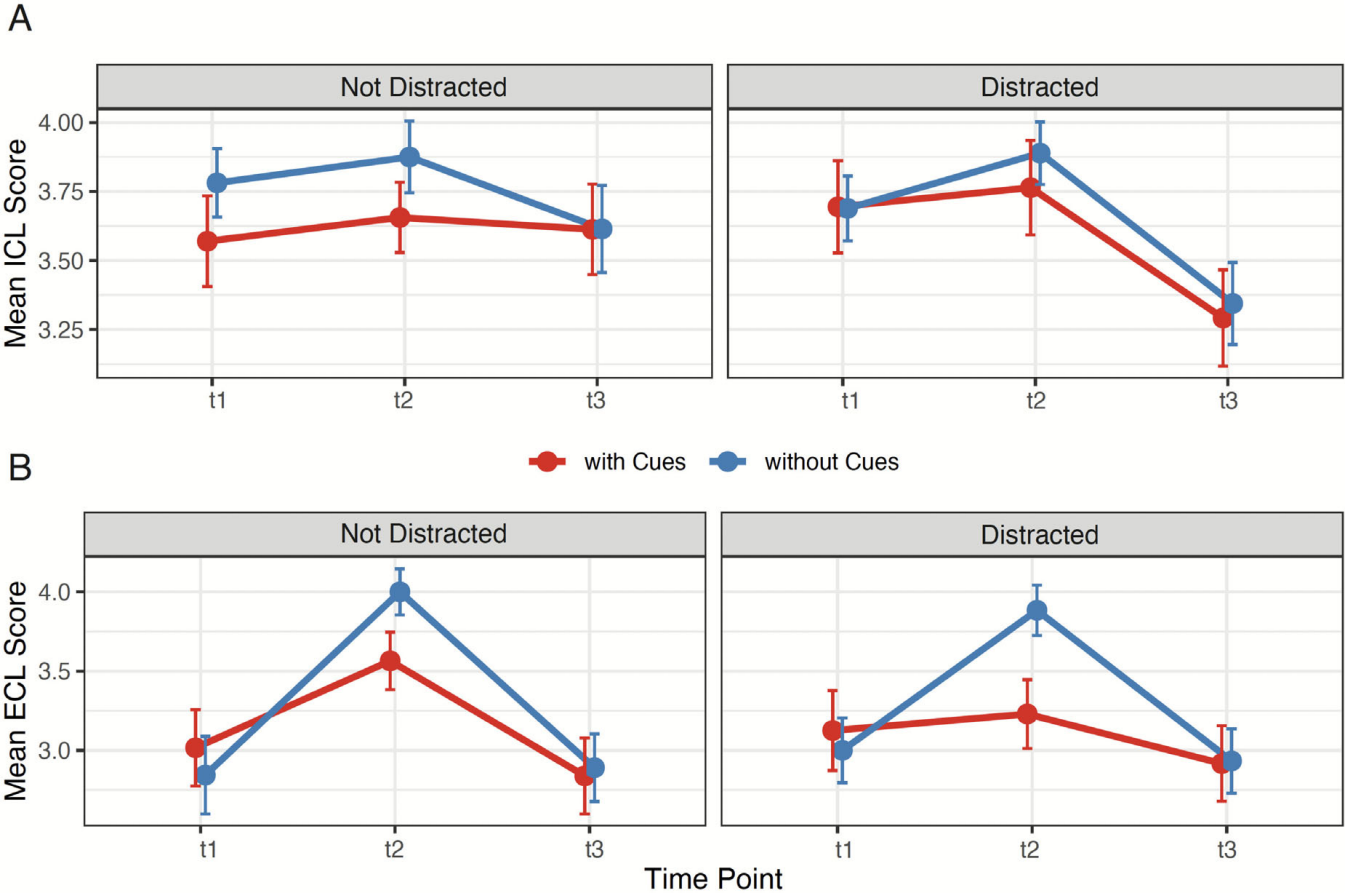
Cueing reduces perceived extraneous cognitive load during training, particularly under distraction. *Mean* intrinsic cognitive load (ICL; panel a) and extraneous cognitive load (ECL; panel b) at pre‐test (t1), during training (t2), and post‐test (t3) for cued (red) and uncued (blue) learners under no‐distraction and distraction conditions. Error bars represent ±1 *standard error* of the *mean*. [Color figure can be viewed at wileyonlinelibrary.com]

During the training phase (t2), cueing demonstrated a selective beneficial effect on cognitive load management. Between‐subjects analyses revealed that cueing significantly reduced ECL compared to the no‐cueing conditions (*F*(1,113) = 9.16, *p* = 0.003, *ηp* ^2^ = 0.075) (Figure 3B). This reduction in ECL occurred without any corresponding effects on ICL, which remained unaffected by cueing (*F*(1,113) = 1.78, *p* = 0.185, *ηp* ^2^ = 0.002) (Figure 3A).

The presence of distraction did not significantly influence either type of cognitive load during training, with no main effects observed for ECL (*F*(1,113) = 1.58, *p* = 0.211, *ηp* ^2^ = 0.014) or ICL (*F*(1,113) = 0.19, *p* = 0.668, *ηp* ^2^ = 0.002). Additionally, no significant interactions were found between cueing and distraction for either ECL (*F*(1,113) = 0.39, *p* = 0.532, *ηp* ^2^ = 0.003) or ICL (*F*(1,113) = 0.12, *p* = 0.727, *ηp* ^2^ = 0.001).

These findings indicate that cueing specifically targets and reduces the extraneous processing demands during learning.

## 4 DISCUSSION

Our findings indicate a load interference pattern: cueing, while effective under undistracted conditions, failed to produce learning benefits when learners' attention was divided by a concurrent distraction task. This suggests that even instructionally sound interventions cannot compensate when available cognitive resources are already monopolised by competing demands, particularly when executive control processes are overtaxed by dual‐task requirements.

In contrast, we observed that when students were undistracted, cueing led to reduced ECL and increased performance. This replicates prior research (e.g. ^5^) that visual cues such as EMME help learners filter relevant from irrelevant information, thereby freeing WM for germane processing. From an attentional perspective, cues appear to facilitate selective attention by redirecting attentional allocation toward task‐relevant features. When an ongoing task is interrupted, goal representations in working memory decay rapidly, requiring costly reactivation once attention returns.^18^, ^19^ We also observed a reduction in ECL without changes in ICL, consistent with CLT, which posits that cueing enhances instructional efficiency without altering the inherent complexity of the material.^20^

However, we found that distraction restricted these benefits. Distracted learners—whether cued or not—showed only marginal performance improvements, with almost identical learning trajectories. This pattern aligns with the *load interference hypothesis*: When the WM system is already taxed by extraneous demands, it cannot allocate enough resources to benefit from additional instructional guidance. Thus, the mental subtraction task likely consumed executive control resources needed to coordinate selective attention, which prevents learning from effectively engaging the attention guidance that cues provide. In other words, cueing requires available capacity to be effective; under distraction, it becomes instructional ‘noise’ that cannot penetrate the bottleneck. From an attentional perspective, distraction may not just consume WM capacity but disrupt the control of selection within WM, and thereby prevent cues from being attended or encoded effectively.^21^

Importantly, considering that CLT treats load types as additive, our data show a nonlinear interference: distraction does not just add ECL but blocks cue benefits almost entirely. This may be theorised as *control load*, which represents the cognitive cost of maintaining task goals and managing interference when executive attention is divided. In this view, distraction imposes an additional layer of control load that competes with the attentional resources required to apply instructional guidance. Once this control load exceeds a certain threshold, the system can no longer sustain both distraction management and instructional cue processing. This threshold‐like collapse resembles cognitive interference patterns observed in applied visual‐search domains such as radiology, where workload increases lead to nonlinear rises in error rates.^22^

Finally and paradoxically, cueing still lowered self‐reported ECL during training even under distraction, yet this did not translate into measurable performance gains. This decoupling between subjective load ratings and performance suggests that learners may feel aided by cues even when they cannot process them effectively. This underscores the limitation of relying solely on self‐reported load measures as proxies for cognitive resource availability.^23^

### 4.1 Implications for medical education theory and practice

Modern medical education environments present multiple competing demands on students' limited working memory: smartphone notifications and social media alerts during lectures; multitasking between electronic health records, clinical decision support systems and patient care during clerkships; simultaneous processing of diagnostic imaging, laboratory results and patient history in clinical reasoning tasks; interruptions from pagers, hospital communication systems and urgent clinical alerts during bedside teaching; and cognitive switching between AI‐assisted diagnostic tools and automated documentation systems.

The proliferation of digital technologies, AI integration and interconnected health care systems means these distractors are steadily increasing in both number and complexity, creating an escalating challenge for cognitive resource management in medical education (see previous works^24^). These distractors create ECL that monopolises the same working memory resources needed for effective learning, rendering even well‐designed instructional interventions ineffective. For example, Radovic et al.^25^ found that even infrequent interruptions in a visual‐search simulation produced marked resumption costs. In practice, this means we need to pair instructional enhancements with systematic distraction control at the curriculum, technology and policy levels.

### 4.2 Device management and distraction control

A blanket ‘no phones’ rule is attractive, but the evidence is mixed. Several quasi‐experimental and review papers report improved focus and, in some contexts, better academic outcomes following restrictions, while others find little effect on grades or well‐being when bans are implemented in isolation, without broader behaviour change or after‐class digital habits shifting (see previous works^26^). Together, these findings support targeted, enforceable, context‐specific restrictions (e.g. bell‐to‐bell or session‐bounded device control) paired with pedagogy and habit training rather than bans alone.^2^ For large‐group didactics and skills training (settings most vulnerable to divided attention) default phone‐off/away policies, lockboxes or signal‐pouches and enforced laptop‐only use for task‐relevant work are justified. Institutions should evaluate not only grades but proximal outcomes (on‐task gaze, note quality and question rates) when assessing policy impact.

Independent of outright bans, notifications themselves impair attention and performance even when devices are not actively used through salience, expectancy and task interruption costs.^27^ Evidence from lab and field studies shows that reducing notification‐caused interruptions improves performance and lowers strain; related work suggests that the mere presence of a smartphone can sap working memory capacity, with partial replications and boundary conditions noted.^28^ For teaching sessions that rely on attentional guidance (e.g. ultrasound or surgery training), we recommend institutional defaults: airplane mode or focus mode mandated at entry, silent‐by‐default campus apps and ‘notification‐blackout’ windows aligned with core teaching blocks. With an increase in proficiency, the amount of distractions should be increased to train for real‐world conditions.

### 4.3 Cognitive load optimization

Our results imply that cueing is most effective when total cognitive load stays below a critical threshold. Educators should therefore make low‐friction design choices that widen the margin for teaching to work: slower pacing, momentary micro‐pauses after salient cues, tiered signalling (few, consistent visual channels) and batching of interactive prompts rather than continuous dual‐task demands.^29^, ^30^ Where devices are necessary (e.g. polling), schedule ‘attention checkpoints’ that explicitly pause nonessential apps. Consider pre‐commitment rituals (‘phones away, focus mode on’) and visible timers that bound concentrated work periods. This extends to assessments: If image interpretation is the target, minimise concurrent digital demands during practice and testing so measured performance reflects learning rather than interruption tolerance.

Importantly, the fast‐paced integration of AI systems into medical training requires careful attention to cognitive load interference.^31^ AI‐powered diagnostic tools, intelligent tutoring systems and clinical decision support must be designed with load interference principles in mind. When learners' cognitive resources are already taxed by AI interfaces and notifications, the primary learning tasks cannot penetrate the WM bottleneck. Institutions should implement AI tools that batch notifications, provide silent background adaptation and offer focused, single‐task learning modes.

Given the pervasiveness of screens and notifications in clinical education (see previous works^32^), students need explicit training in *attention management* as part of digital professionalism. Short, skills‐based modules can cover: configuring focus modes, batching notifications, single‐tasking protocols during critical learning and reflective tracking of attention failures. This complements policy and avoids framing the issue as purely punitive. Programmes could teach ‘clinical attention hygiene’ alongside handovers and situational awareness. In sum, our findings suggest that instructional elements in medical education should be paired with explicit load management, rather than expected to compensate for environments that already exhaust learners' cognitive resources.

### 4.4 Limitations and future directions

Several limitations should be considered when interpreting these findings. First, our distraction paradigm employed a standardised mental arithmetic task that may not fully capture the ecological complexity of real‐world (extraneous) distractions. While this simple approach ensured experimental control and replicability, actual clinical environments present more heterogeneous interruptions, varying in timing, modality, urgency and cognitive demands. Future studies should examine load interference effects using more naturalistic distraction paradigms, such as simulated clinical communication interruptions or authentic multitasking scenarios encountered during clerkships. Future work should also consider dynamic models of interruption and goal resumption to capture how temporal gaps in attention affect the continuity of learning processes.

In addition, the measurement of cognitive load through self‐report scales, while validated, may not capture the full complexity of resource allocation during dual‐task performance. Future investigations should incorporate physiological measures (eye‐tracking, EEG and heart rate variability) or other secondary‐task performance indicators to provide more objective indices of cognitive resource depletion.

Several promising directions emerge for future research. First, investigating individual differences in *distraction susceptibility* could inform personalised approaches to attention management training. Factors such as WM capacity, attentional control and prior clinical experience may moderate load interference effects, suggesting that some learners might benefit more from distraction‐control interventions than others.

Second, research will examine the temporal dynamics of load interference, how quickly cognitive resources become unavailable under distraction, whether brief respites can restore cueing effectiveness and what recovery periods are needed between competing cognitive demands. This could inform optimal scheduling of high‐attention learning activities and the design of ‘cognitive break’ protocols.

Third, the development and testing of attention‐aware educational technologies represent a critical frontier. Adaptive systems that monitor learner cognitive load in real time and automatically adjust instructional complexity, reduce notifications or suggest breaks could help maintain the cognitive conditions necessary for effective learning. However, such systems must be carefully designed to avoid creating additional cognitive burdens through their monitoring and intervention mechanisms.

## 5 CONCLUSION

Our findings demonstrate a cognitive interference effect: even well‐designed instructional interventions like cueing cannot compensate when learners' cognitive resources are monopolised by distraction.

This load interference mechanism has profound implications for medical education in an increasingly connected world, where digital distractions are proliferating rapidly. Rather than relying solely on improved instructional design, medical educators must engineer learning environments that protect cognitive resources through systematic distraction control, attention management training and technology design principles that prioritise focused learning over constant connectivity. The future of medical education depends not just on *how* we teach, but on creating conditions where effective learning can unfold.

## AUTHOR CONTRIBUTIONS

**Andrea Storck:** Conceptualization; writing—original draft; writing—review and editing. **Clemens Grahl Römer:** Investigation; Data Curation; writing—review and editing. **Steffen Ansorge:** methodology; software. **Eva Schönefeld:** validation; supervision. **Michelle Bellstedt:** validation; resources. **Birte Barbian:** writing—review and editing. **Martin Janssen:** validation; supervision. **Konstantin E. Seifert:** validation; project administration. **Dogus Darici:** Conceptualization; methodology; software; data curation; investigation; validation; formal analysis; supervision; resources; writing—review and editing; writing—original draft; visualization; project administration.

## CONFLICT OF INTEREST STATEMENT

The authors declare no conflicts of interest.

## ETHICS STATEMENT

The study was reviewed by the ethics committee of the university (“Ethik Kommission Westfalen‐Lippe”) and deemed not to require formal medical ethics approval (reference: 2023‐631‐f‐N).

## AI DISCLOSURE

Claude v. 4 Sonnet and GPT‐5 have been used for language editing. All content and ideas remain the original work of the authors, with AI assistance to improve linguistic clarity.

## Supporting information


**Table S1.** Bayes statistics for informative hypothesis testing using the R package bain. Effects on post‐pre‐performance are computed.
**Figure S1.** Stimuli to assess pre‐ and posttest performance.
**Table S2.** Item analysis.

## Data Availability

The data that support the findings of this study are available from the corresponding author upon reasonable request.
