# Supplementary material for: Learning and distraction: Evidence for cognitive load interference in medical education
Source: Med Educ. 2025 Dec 18;60(6):676–84. doi: 10.1111/medu.70136 (PMC13129625; doi:10.1111/medu.70136)
Supplement: Supplementary file 1 — Table S1. Bayes statistics for informative hypothesis testing using the R package bain. Effects on post‐pre‐performance are computed. Figure S1. Stimuli to assess pre‐ and posttest performance. Table S2. Item analysis. [file MEDU-60-676-s001.docx]

**Learning and distraction:
Evidence for cognitive load interference in medical education**

**Keywords:** Medical education, distraction, cognitive load, cognitive load interference, learning performance, eye tracking

**Short title:** Learning and distraction

**Acknowledgements:** We thank all the students for participating in this study.

**Conflicts of interest:** The authors declare no conflicts of interest.

**AI disclosure:** Claude v. 4 Sonnet and GPT-5 have been used for language editing. All content and ideas remain the original work of the authors, with AI assistance to improve linguistic clarity.

**Funding:** This research did not receive any specific grant from funding agencies in the public, commercial, or not-for-profit sectors.

**Bayes statistics**

| **Supplemental Table 1**  Bayes statistics for informative hypothesis testing using the R packge bain. Effects on post-pre performance are computed. | | | | | |
| --- | --- | --- | --- | --- | --- |
| Hypothesis | Model | *Fit* | *Complexity* | *Bayes Factor* | *Posterior Model Propability* |
| 1) no interference | G1-G2=G3-G4 | 0.038 | 0.046 | 0.841 | 0.457 |
| 2) high interference | G3=G4 | 0.541 | 0.062 | 8.698 | 0.897 |
| 3) partial interference | G1>G3&G3>G4 | 0.746 | 0.152 | 4.911 | 0.831 |
| *Note. The model specifications were derived from the hypotheses 1-3. The Bayes factor and the Posterior Model Propability provide highest support for the high interference hypothesis.*  *Abbreviations. G1 = Cueing + No Distraction; G2 = No Cueing + No Distraction; G3 = Cueing + Distraction, G4 = No Cueing + Distraction.* | | | | | |

**Items to assess prior knowledge in anatomy**

Bitte füllen Sie die folgenden Fragen aus.

1) Welche Aussage zur regelhaften topographischen Lage des Magens trifft zu?

1. Der Magenfundus berührt beim stehenden Menschen das Centrum tendineum des Zwerchfells.
2. Der größte Teil der Vorderwand des Magens wird vom Lobus caudatus der Leber bedeckt.
3. Der kaudalste Punkt des Magens befindet sich am Übergang ins Duodenum (Canalis pyloricus).
4. Die Hinterwand des Magens grenzt an das Mesenterium.
5. Die Cardia befindet sich im Hiatus oesophageus des Zwerchfells.

2) Welches Lebersegment ist dem Lobus caudatus zugeordnet?

1. I
2. II
3. III
4. IV
5. V

3) Welche der aufgeführten Impressionen befindet sich am linken Leberlappen (Lobus hepaticus sinister)?

1. Impressio colica
2. Impressio duodenalis
3. Impressio gastrica
4. Impressio renalis
5. Impressio suprarenalis

4) In welchen Abschnitt des Duodenums mündet der Ductus choledochus?

1. Bulbus
2. Pars superior
3. Pars descendens
4. Pars transversa
5. Pars ascendens

5) Die Milznische wird kaudal (unten) begrenzt durch das

1. Lig. gastrocolicum.
2. Lig. gastrosplenicum.
3. Lig. phrenicocolicum.
4. Lig. splenorenale.
5. Mesocolon transversum.

6) Die Capsula adiposa der Niere

1. ist typischerweise lateral offen.
2. liegt innerhalb der Capsula fibrosa renis.
3. liegt typischerweise dorsal des hinteren Blatts der Fascia renalis.
4. liegt typischerweise ventral des vorderen Blatts der Fascia renalis.
5. umgibt auch die Nebenniere.

7) Das Blut der V. gastroomentalis dextra fließt im Regelfall in die

1. V. gastrica dextra.
2. V. gastrica sinistra.
3. V. mesenterica inferior.
4. V. mesenterica superior.
5. V. splenica.

8) Welche Gefäße bilden am wahrscheinlichsten eine Verbindung zwischen dem Gefäßgebiet der A. mesenterica superior und dem der A. mesenterica inferior?

1. A. rectalis superior und Aa. sigmoideae
2. A. gastroduodenalis und A. ileocolica
3. A. colica media und A. colica sinistra
4. A. colica dextra und A. colica sinistra
5. A. pancreatica magna und A. pancreaticoduodenalis inferior

9) Bei einer Operation im Oberbauch haben Sie das Omentum minus durchtrennt und legen eine Arterie frei, die von der A. hepatica communis nach kaudal entspringt und direkt ventral des Caput pancreatis aus Ihrem Gesichtsfeld verschwindet. Hierbei handelt es sich um die

1. A. hepatica propria.
2. A. gastrica dextra.
3. A. gastroduodenalis.
4. A. gastrica sinistra.
5. A. pancreaticoduodenalis superior posterior.

10) Eine portokavale Anastomose kann sich am ehesten bilden unter Beteiligung der

1. V. gastrica sinistra.
2. V. gastroomentalis dextra.
3. V. sigmoidea.
4. V. splenica.
5. V. testicularis.

**Supplemental Figure 1**

Stimuli to assess pre- and posttest performance

**
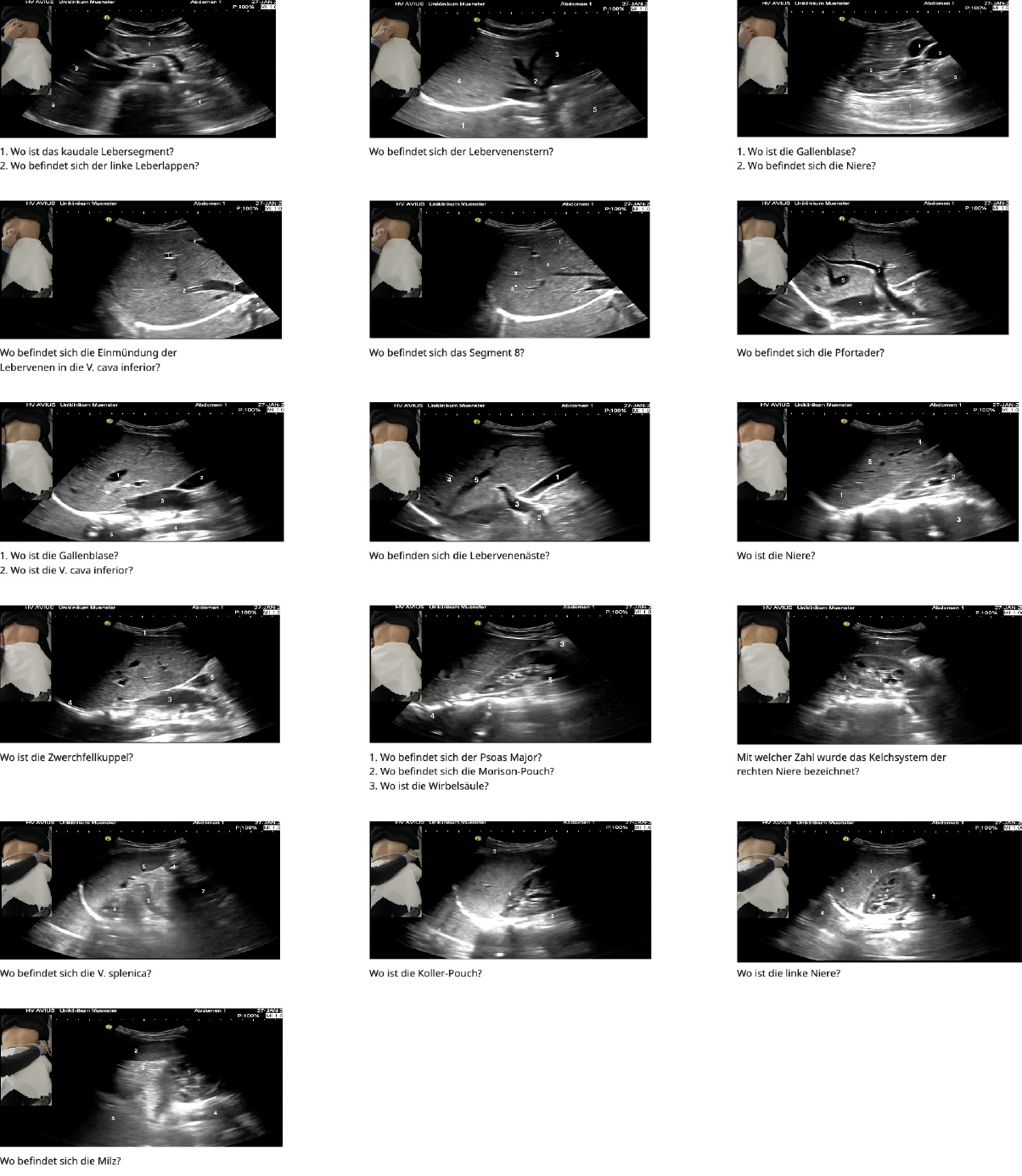
**

**Item analysis**

| Supplemental Table 2  **Item analysis** | | | | | | | |
| --- | --- | --- | --- | --- | --- | --- | --- |
| Item | *N* | *Mean* | *SD* | *Difficulty* | *Discriminatory* | *CI95% lower* | *CI95% upper* |
| pre1 | 118 | 0.288 | 0.455 | 0.288 | 0.102 | 0.206 | 0.370 |
| pre2 | 118 | 0.381 | 0.488 | 0.381 | 0.045 | 0.294 | 0.469 |
| pre3 | 118 | 0.932 | 0.252 | 0.932 | 0.262 | 0.887 | 0.978 |
| pre4 | 118 | 0.398 | 0.492 | 0.398 | 0.205 | 0.310 | 0.487 |
| pre5 | 118 | 0.178 | 0.384 | 0.178 | 0.392 | 0.109 | 0.247 |
| pre6 | 118 | 0.186 | 0.391 | 0.186 | 0.052 | 0.116 | 0.257 |
| pre7 | 118 | 0.127 | 0.335 | 0.127 | 0.144 | 0.067 | 0.187 |
| pre8 | 118 | 0.458 | 0.500 | 0.458 | 0.084 | 0.368 | 0.548 |
| pre9 | 118 | 0.500 | 0.502 | 0.500 | 0.203 | 0.410 | 0.590 |
| pre10 | 118 | 0.686 | 0.466 | 0.686 | 0.294 | 0.603 | 0.770 |
| pre11 | 118 | 0.322 | 0.469 | 0.322 | 0.403 | 0.238 | 0.406 |
| pre12 | 118 | 0.585 | 0.495 | 0.585 | 0.176 | 0.496 | 0.674 |
| pre14 | 118 | 0.229 | 0.422 | 0.229 | 0.092 | 0.153 | 0.305 |
| pre15 | 118 | 0.695 | 0.462 | 0.695 | 0.231 | 0.612 | 0.778 |
| pre16 | 118 | 0.415 | 0.495 | 0.415 | 0.182 | 0.326 | 0.504 |
| pre17 | 118 | 0.373 | 0.486 | 0.373 | 0.226 | 0.286 | 0.460 |
| pre18 | 118 | 0.847 | 0.361 | 0.847 | 0.283 | 0.783 | 0.912 |
| pre19 | 118 | 0.449 | 0.500 | 0.449 | 0.260 | 0.359 | 0.539 |
| pre20 | 118 | 0.441 | 0.499 | 0.441 | 0.098 | 0.351 | 0.530 |
| post1 | 118 | 0.364 | 0.483 | 0.364 | 0.051 | 0.278 | 0.451 |
| post2 | 118 | 0.559 | 0.499 | 0.559 | 0.120 | 0.470 | 0.649 |
| post4 | 118 | 0.381 | 0.488 | 0.381 | 0.225 | 0.294 | 0.469 |
| post5 | 118 | 0.475 | 0.501 | 0.475 | 0.196 | 0.384 | 0.565 |
| post6 | 118 | 0.136 | 0.344 | 0.136 | 0.228 | 0.074 | 0.197 |
| post7 | 118 | 0.144 | 0.353 | 0.144 | 0.106 | 0.081 | 0.207 |
| post8 | 118 | 0.483 | 0.502 | 0.483 | 0.151 | 0.393 | 0.573 |
| post9 | 118 | 0.559 | 0.499 | 0.559 | 0.173 | 0.470 | 0.649 |
| post10 | 118 | 0.729 | 0.446 | 0.729 | 0.289 | 0.649 | 0.809 |
| post11 | 118 | 0.746 | 0.437 | 0.746 | 0.233 | 0.667 | 0.824 |
| post12 | 118 | 0.653 | 0.478 | 0.653 | 0.125 | 0.567 | 0.738 |
| post14 | 118 | 0.322 | 0.469 | 0.322 | 0.020 | 0.238 | 0.406 |
| post15 | 118 | 0.780 | 0.416 | 0.780 | 0.131 | 0.705 | 0.854 |
| post16 | 118 | 0.576 | 0.496 | 0.576 | 0.254 | 0.487 | 0.665 |
| post17 | 118 | 0.551 | 0.500 | 0.551 | 0.283 | 0.461 | 0.641 |
| post18 | 118 | 0.915 | 0.280 | 0.915 | 0.332 | 0.865 | 0.966 |
| post19 | 118 | 0.525 | 0.501 | 0.525 | 0.132 | 0.435 | 0.616 |
| post20 | 118 | 0.500 | 0.502 | 0.500 | 0.128 | 0.410 | 0.590 |

**Compiled report of the R script**

library(dplyr)

##
## Attaching package: 'dplyr'

## The following objects are masked from 'package:stats':
##
## filter, lag

## The following objects are masked from 'package:base':
##
## intersect, setdiff, setequal, union

library(tidyr)
library(ggplot2)
library(gridExtra)

##
## Attaching package: 'gridExtra'

## The following object is masked from 'package:dplyr':
##
## combine

library(psych)

##
## Attaching package: 'psych'

## The following objects are masked from 'package:ggplot2':
##
## %+%, alpha

d <- read.csv("/Users/XXXX/Desktop/Distraction_EMME/data/Cueing_Results.csv")

# data cleaning: create scales, filter relevant scales, omit item-level data etc.
d$ultrasound_prkno <- rowMeans(d[, c("US_anat_identification", "US_interpretation", "preknowledge")],
 na.rm = TRUE)
d$page_flow <- factor(d$page_flow,
 levels = c("noDistractionsNoCueing",
 "distractionsNoCueing",
 "noDistractionsCueing",
 "distractionsCueing"))
d <- d %>%
 separate(page_flow, into = c("distraction", "cueing"), sep = "(?<=Distractions|distractions)(?=Cueing|NoCueing)", remove = FALSE)

d1<- d %>%
 select(page_flow,
 distraction,
 cueing,
 age,
 gender,
 ultrasound_prkno,
 preparation,
 SUM_ANATOMY_PERFORMANCE,
 SUM_PRE_TIME,
 SUM_POST_TIME,
 SUM_PRE_PERFORMANCE,
 SUM_POST_PERFORMANCE,
 ICL_t1,
 ICL_t2,
 ICL_t3,
 ECL_t1,
 ECL_t2,
 ECL_t3)%>%
 mutate(
 gender = recode(as.factor(gender), `1` = "male", `2` = "female"),
 cueing = as.factor(cueing),
 page_flow = as.factor(page_flow),
 distraction = as.factor(distraction),
 across(!c(gender, page_flow, distraction, cueing), ~ as.numeric(.)
 )
 )
summary(d1)

## page_flow distraction cueing age
## noDistractionsNoCueing:32 distractions :55 Cueing :55 Min. :19.00
## distractionsNoCueing :31 noDistractions:63 NoCueing:63 1st Qu.:20.00
## noDistractionsCueing :31 Median :20.00
## distractionsCueing :24 Mean :21.08
## 3rd Qu.:21.00
## Max. :40.00
## gender ultrasound_prkno preparation SUM_ANATOMY_PERFORMANCE
## male :42 Min. :1.000 Min. :1.000 Min. :1.000
## female:76 1st Qu.:1.333 1st Qu.:1.000 1st Qu.:3.000
## Median :1.667 Median :2.000 Median :4.000
## Mean :1.777 Mean :2.034 Mean :4.695
## 3rd Qu.:2.250 3rd Qu.:3.000 3rd Qu.:6.000
## Max. :3.333 Max. :4.000 Max. :9.000
## SUM_PRE_TIME SUM_POST_TIME SUM_PRE_PERFORMANCE SUM_POST_PERFORMANCE
## Min. : 151440 Min. :109572 Min. : 2.000 Min. : 4.00
## 1st Qu.: 322849 1st Qu.:163844 1st Qu.: 7.000 1st Qu.: 8.25
## Median : 385734 Median :209016 Median : 8.000 Median :10.00
## Mean : 412114 Mean :217023 Mean : 8.492 Mean :10.37
## 3rd Qu.: 467180 3rd Qu.:262248 3rd Qu.:10.000 3rd Qu.:12.00
## Max. :1313248 Max. :383583 Max. :14.000 Max. :16.00
## ICL_t1 ICL_t2 ICL_t3 ECL_t1
## Min. :1.000 Min. :1.000 Min. :1.000 Min. :1.000
## 1st Qu.:3.333 1st Qu.:3.333 1st Qu.:3.000 1st Qu.:2.000
## Median :3.667 Median :4.000 Median :3.500 Median :3.000
## Mean :3.689 Mean :3.788 Mean :3.489 Mean :2.996
## 3rd Qu.:4.333 3rd Qu.:4.333 3rd Qu.:4.000 3rd Qu.:4.000
## Max. :5.000 Max. :5.000 Max. :5.000 Max. :5.000
## ECL_t2 ECL_t3
## Min. :1.000 Min. :1.000
## 1st Qu.:3.000 1st Qu.:2.000
## Median :4.000 Median :2.750
## Mean :3.682 Mean :2.894
## 3rd Qu.:4.500 3rd Qu.:4.000
## Max. :5.000 Max. :5.000

# overview, check data quality
hist(d1$SUM_ANATOMY_PERFORMANCE)


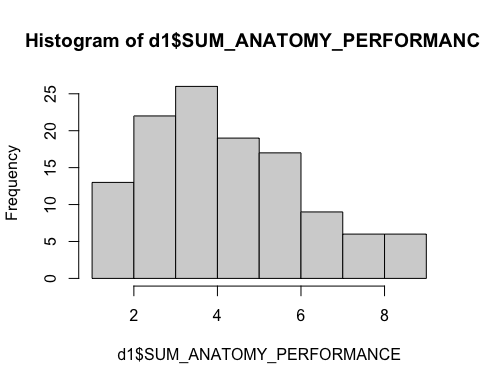


hist(d1$SUM_PRE_TIME) # one outlier detected


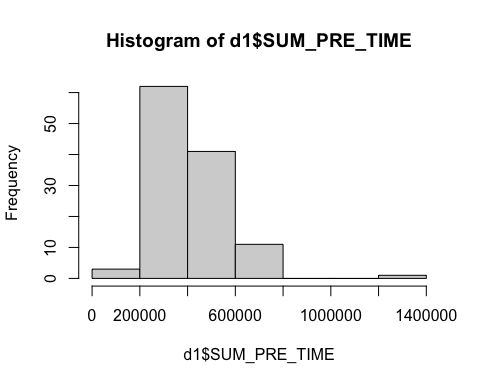


boxplot(d1$SUM_PRE_TIME) # one outlier confirmed


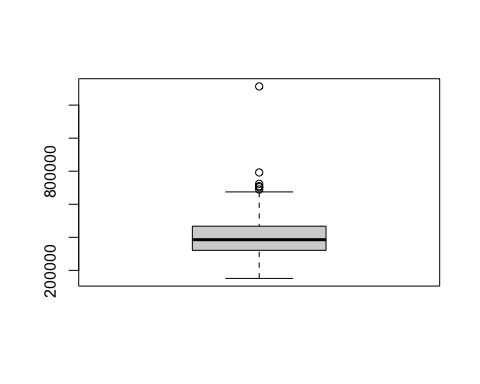


d2 <- d1[-118,] # outlier in row 118 excluded (distractionNoCueing group)
hist(d2$SUM_PRE_TIME) # check after exclusion looks good


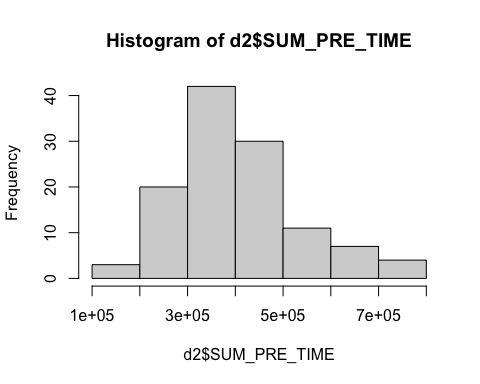


hist(d2$SUM_POST_TIME)


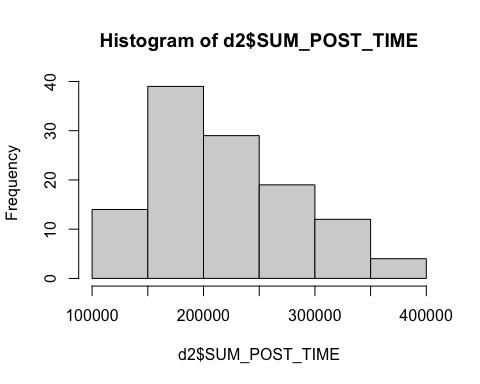


hist(d2$SUM_PRE_PERFORMANCE)


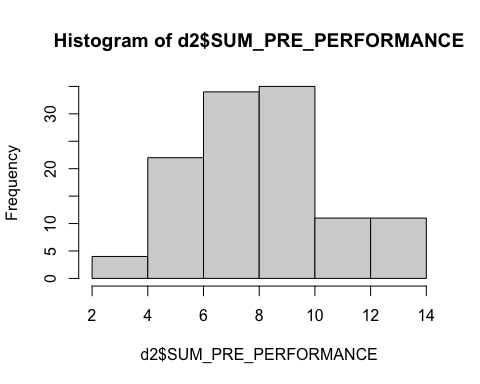


hist(d2$SUM_POST_PERFORMANCE)


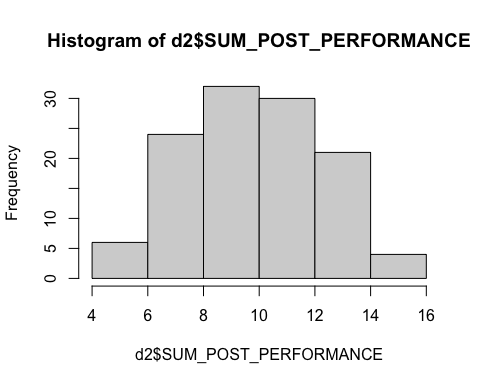


hist(d2$ICL_t1)


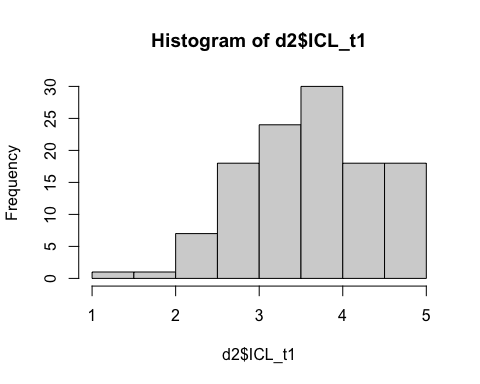


hist(d2$ICL_t2) # low variance


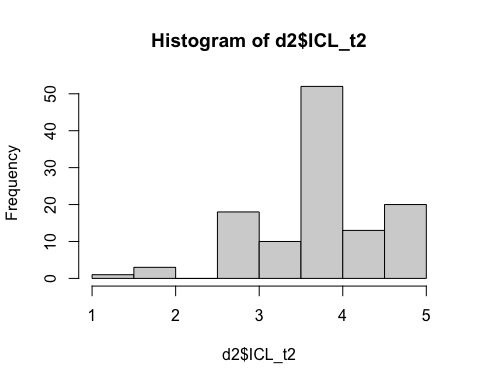


hist(d2$ICL_t3)


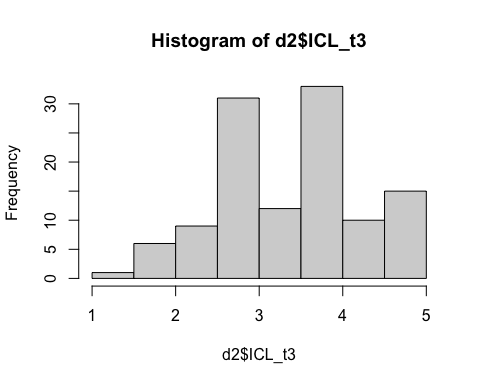


hist(d2$ECL_t1) # bimodal distribution


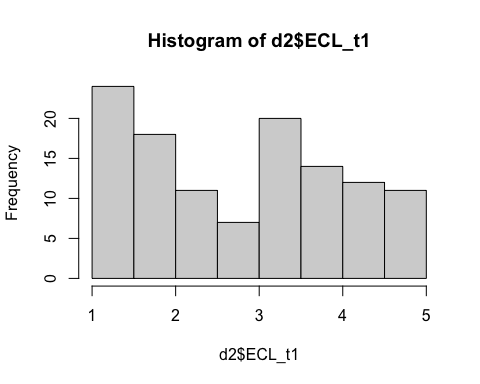


hist(d2$ECL_t2)


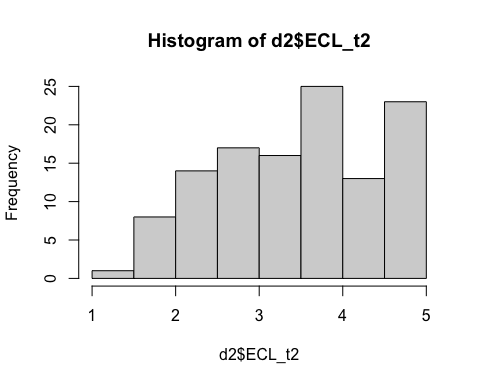


hist(d2$ECL_t3)


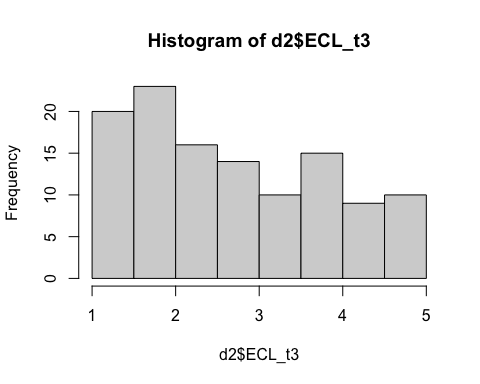


# repeated measures anova for performance (pre vs. post for distraction vs. cueing)
d2$subject_id <- seq_len(nrow(d2))
d_long <- pivot_longer(d2,
 cols = c(SUM_PRE_PERFORMANCE, SUM_POST_PERFORMANCE),
 names_to = "time",
 values_to = "performance")
d_long$time <- factor(d_long$time, levels = c("SUM_PRE_PERFORMANCE", "SUM_POST_PERFORMANCE"))
d_long$cueing <- factor(d_long$cueing)
d_long$distraction <- factor(d_long$distraction)
anova_result <- aov(performance ~ time * cueing * distraction +
 Error(subject_id/time), data = d_long)
summary(anova_result)

##
## Error: subject_id
## Df Sum Sq Mean Sq
## cueing 1 0.6206 0.6206
##
## Error: subject_id:time
## Df Sum Sq Mean Sq
## time 1 150.4 150.4
##
## Error: Within
## Df Sum Sq Mean Sq F value Pr(>F)
## time 1 56.6 56.59 9.228 0.00267 **
## cueing 1 1.1 1.09 0.177 0.67400
## distraction 1 4.2 4.16 0.678 0.41124
## time:cueing 1 31.1 31.09 5.070 0.02531 *
## time:distraction 1 44.0 44.00 7.174 0.00795 **
## cueing:distraction 1 39.6 39.63 6.463 0.01169 *
## time:cueing:distraction 1 12.2 12.21 1.991 0.15964
## Residuals 224 1373.7 6.13
## ---
## Signif. codes: 0 '***' 0.001 '**' 0.01 '*' 0.05 '.' 0.1 ' ' 1

# repeated measures anova for ecl and icl (pre, during, post for distraction vs. cueing)
d2$subject_id <- seq_len(nrow(d2))
d_long <- pivot_longer(d2,
 cols = c(SUM_PRE_PERFORMANCE, SUM_POST_PERFORMANCE),
 names_to = "time",
 values_to = "performance")
d_long$time <- factor(d_long$time, levels = c("SUM_PRE_PERFORMANCE", "SUM_POST_PERFORMANCE"))
d_long$cueing <- factor(d_long$cueing)
d_long$distraction <- factor(d_long$distraction)
anova_result <- aov(performance ~ time * cueing * distraction +
 Error(subject_id/time), data = d_long)
summary(anova_result)

##
## Error: subject_id
## Df Sum Sq Mean Sq
## cueing 1 0.6206 0.6206
##
## Error: subject_id:time
## Df Sum Sq Mean Sq
## time 1 150.4 150.4
##
## Error: Within
## Df Sum Sq Mean Sq F value Pr(>F)
## time 1 56.6 56.59 9.228 0.00267 **
## cueing 1 1.1 1.09 0.177 0.67400
## distraction 1 4.2 4.16 0.678 0.41124
## time:cueing 1 31.1 31.09 5.070 0.02531 *
## time:distraction 1 44.0 44.00 7.174 0.00795 **
## cueing:distraction 1 39.6 39.63 6.463 0.01169 *
## time:cueing:distraction 1 12.2 12.21 1.991 0.15964
## Residuals 224 1373.7 6.13
## ---
## Signif. codes: 0 '***' 0.001 '**' 0.01 '*' 0.05 '.' 0.1 ' ' 1

# Create summary statistics for plotting
d_summary <- d_long %>%
 group_by(time, cueing, distraction) %>%
 summarise(
 mean_performance = mean(performance, na.rm = TRUE),
 sd_performance = sd(performance, na.rm = TRUE),
 se_performance = sd_performance / sqrt(n()),
 n = n(),
 .groups = 'drop'
 )

# 1. Main interaction plot: Time x Cueing x Distraction
p1 <- ggplot(d_summary, aes(x = time, y = mean_performance,
 color = cueing, group = cueing)) +
 geom_line(size = 1.2, position = position_dodge(0.1)) +
 geom_point(size = 3, position = position_dodge(0.1)) +
 geom_errorbar(aes(ymin = mean_performance - se_performance,
 ymax = mean_performance + se_performance),
 width = 0.1, position = position_dodge(0.1)) +
 facet_wrap(~ factor(distraction, levels = c("noDistractions", "distractions")),
 labeller = label_both) +
 scale_x_discrete(labels = c("Pre", "Post")) +
 scale_color_brewer(type = "qual", palette = "Set1") +
 labs(title = "Performance Over Time by Cueing and Distraction Conditions",
 subtitle = "Error bars represent standard error",
 x = "Time Point",
 y = "Mean Performance",
 color = "Cueing Condition") +
 theme_bw() +
 theme(axis.text.x = element_text(angle = 45, hjust = 1),
 plot.title = element_text(hjust = 0.5),
 plot.subtitle = element_text(hjust = 0.5))

## Warning: Using `size` aesthetic for lines was deprecated in ggplot2 3.4.0.
## ℹ Please use `linewidth` instead.
## This warning is displayed once every 8 hours.
## Call `lifecycle::last_lifecycle_warnings()` to see where this warning was
## generated.

# 2. Box plots showing distributions
p2 <- ggplot(d_long, aes(x = time, y = performance, fill = cueing)) +
 geom_boxplot(position = position_dodge(0.8), alpha = 0.7) +
 facet_wrap(~ distraction, labeller = label_both) +
 scale_x_discrete(labels = c("Pre", "Post")) +
 scale_fill_brewer(type = "qual", palette = "Set2") +
 labs(title = "Distribution of Performance Scores",
 x = "Time Point",
 y = "Performance",
 fill = "Cueing Condition") +
 theme_bw() +
 theme(plot.title = element_text(hjust = 0.5))

# 3. Individual trajectories plot (spaghetti plot) - grouped by distraction condition
p3 <- ggplot(d_long, aes(x = time, y = performance, group = subject_id)) +
 geom_line(aes(color = cueing), alpha = 0.4) +
 geom_smooth(aes(group = cueing, color = cueing),
 method = "lm", se = TRUE, size = 1.5, alpha = 0.8) +
 facet_wrap(~ distraction, labeller = label_both) +
 scale_x_discrete(labels = c("Pre", "Post")) +
 scale_color_brewer(type = "qual", palette = "Set1") +
 labs(title = "Individual Trajectories by Distraction and Cueing Conditions",
 subtitle = "Individual lines and group trends colored by cueing condition",
 x = "Time Point",
 y = "Performance",
 color = "Cueing Condition") +
 theme_bw() +
 theme(plot.title = element_text(hjust = 0.5),
 plot.subtitle = element_text(hjust = 0.5))

# 4. Change scores (Post - Pre) by conditions
d_change <- d2 %>%
 mutate(change_score = SUM_POST_PERFORMANCE - SUM_PRE_PERFORMANCE)

p4 <- ggplot(d_change, aes(x = distraction, y = change_score, fill = cueing)) +
 geom_boxplot(position = position_dodge(0.8), alpha = 0.7) +
 geom_hline(yintercept = 0, linetype = "dashed", color = "red") +
 scale_fill_brewer(type = "qual", palette = "Pastel1") +
 labs(title = "Change Scores (Post - Pre) by Experimental Conditions",
 subtitle = "Dashed line at zero indicates no change",
 x = "Distraction Condition",
 y = "Change Score (Post - Pre)",
 fill = "Cueing Condition") +
 theme_bw() +
 theme(plot.title = element_text(hjust = 0.5),
 plot.subtitle = element_text(hjust = 0.5),
 axis.text.x = element_text(angle = 45, hjust = 1))

# 5. Bar plot with error bars
p5 <- ggplot(d_summary, aes(x = time, y = mean_performance, fill = cueing)) +
 geom_bar(stat = "identity", position = position_dodge(0.9), alpha = 0.8) +
 geom_errorbar(aes(ymin = mean_performance - se_performance,
 ymax = mean_performance + se_performance),
 position = position_dodge(0.9), width = 0.3) +
 facet_wrap(~ distraction, labeller = label_both) +
 scale_x_discrete(labels = c("Pre", "Post")) +
 scale_fill_brewer(type = "qual", palette = "Set3") +
 labs(title = "Mean Performance by Condition",
 x = "Time Point",
 y = "Mean Performance",
 fill = "Cueing Condition") +
 theme_bw() +
 theme(plot.title = element_text(hjust = 0.5))

# Display all plots
print(p1)


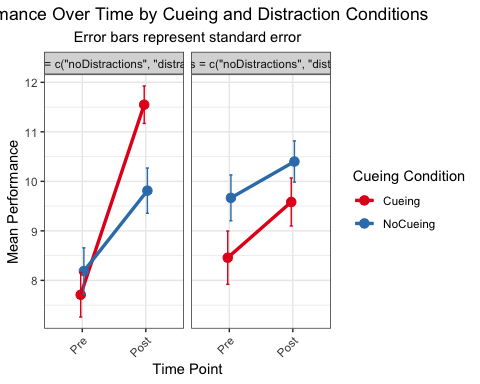


print(p2)


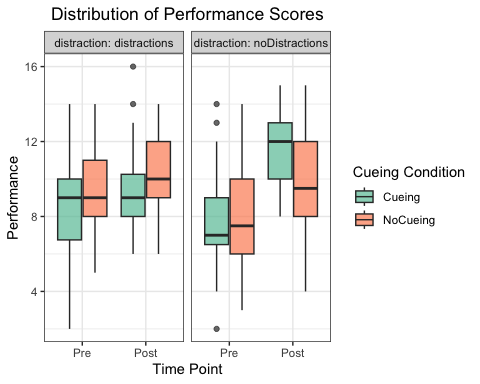


print(p3)

## `geom_smooth()` using formula = 'y ~ x'


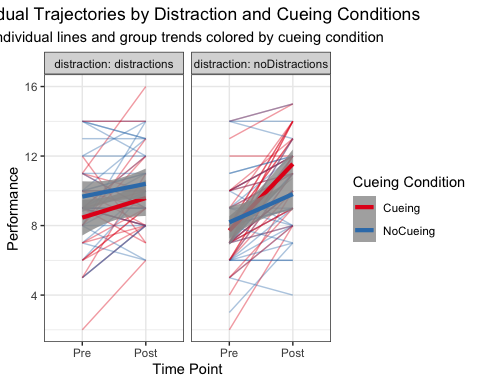


print(p4)


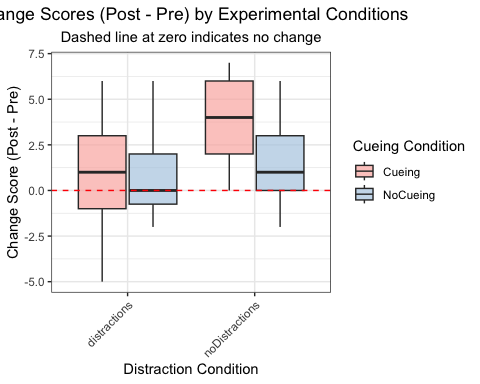


print(p5)


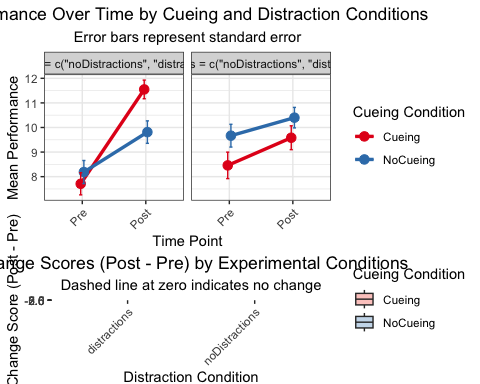


# Print statistics

print("Summary Statistics by Condition:")

## [1] "Summary Statistics by Condition:"

print(d_summary)

## # A tibble: 8 × 7
## time cueing distraction mean_performance sd_performance se_performance n
## <fct> <fct> <fct> <dbl> <dbl> <dbl> <int>
## 1 SUM_P… Cueing distractio… 8.46 2.64 0.538 24
## 2 SUM_P… Cueing noDistract… 7.71 2.51 0.450 31
## 3 SUM_P… NoCue… distractio… 9.67 2.54 0.463 30
## 4 SUM_P… NoCue… noDistract… 8.19 2.66 0.470 32
## 5 SUM_P… Cueing distractio… 9.58 2.38 0.485 24
## 6 SUM_P… Cueing noDistract… 11.5 2.11 0.379 31
## 7 SUM_P… NoCue… distractio… 10.4 2.28 0.417 30
## 8 SUM_P… NoCue… noDistract… 9.81 2.58 0.457 32

# Additional analysis: change scores
effect_sizes <- d_change %>%
 group_by(cueing, distraction) %>%
 summarise(
 mean_change = mean(change_score, na.rm = TRUE),
 sd_change = sd(change_score, na.rm = TRUE),
 n = n(),
 cohen_d = mean_change / sd_change,
 .groups = 'drop'
 )

print("Effect Sizes (Cohen's d) for Change Scores:")

## [1] "Effect Sizes (Cohen's d) for Change Scores:"

print(effect_sizes)

## # A tibble: 4 × 6
## cueing distraction mean_change sd_change n cohen_d
## <fct> <fct> <dbl> <dbl> <int> <dbl>
## 1 Cueing distractions 1.12 2.76 24 0.408
## 2 Cueing noDistractions 3.84 2.02 31 1.90
## 3 NoCueing distractions 0.733 1.96 30 0.373
## 4 NoCueing noDistractions 1.62 1.98 32 0.821

######### COGNITIVE LOAD ANALYSIS

# Create long format data for ICL measures
icl_long <- d2 %>%
 select(subject_id, page_flow, distraction, cueing, ICL_t1, ICL_t2, ICL_t3) %>%
 pivot_longer(cols = c(ICL_t1, ICL_t2, ICL_t3),
 names_to = "time_point",
 values_to = "ICL_score") %>%
 mutate(time_point = factor(time_point, levels = c("ICL_t1", "ICL_t2", "ICL_t3")))

# Create long format data for ECL measures
ecl_long <- d2 %>%
 select(subject_id, page_flow, distraction, cueing, ECL_t1, ECL_t2, ECL_t3) %>%
 pivot_longer(cols = c(ECL_t1, ECL_t2, ECL_t3),
 names_to = "time_point",
 values_to = "ECL_score") %>%
 mutate(time_point = factor(time_point, levels = c("ECL_t1", "ECL_t2", "ECL_t3")))

# Summary statistics for ICL
icl_summary <- icl_long %>%
 group_by(time_point, cueing, distraction) %>%
 summarise(
 mean_icl = mean(ICL_score, na.rm = TRUE),
 sd_icl = sd(ICL_score, na.rm = TRUE),
 se_icl = sd_icl / sqrt(n()),
 n = n(),
 .groups = 'drop'
 )

# Summary statistics for ECL
ecl_summary <- ecl_long %>%
 group_by(time_point, cueing, distraction) %>%
 summarise(
 mean_ecl = mean(ECL_score, na.rm = TRUE),
 sd_ecl = sd(ECL_score, na.rm = TRUE),
 se_ecl = sd_ecl / sqrt(n()),
 n = n(),
 .groups = 'drop'
 )

# SPLIT COGNITIVE LOAD INTO PHASES

# Long format for ICL & ECL
icl_long <- d2 %>%
 select(subject_id, page_flow, distraction, cueing, ICL_t1, ICL_t2, ICL_t3) %>%
 pivot_longer(cols = c(ICL_t1, ICL_t2, ICL_t3),
 names_to = "time_point",
 values_to = "ICL_score") %>%
 mutate(time_point = factor(time_point,
 levels = c("ICL_t1", "ICL_t2", "ICL_t3"),
 labels = c("t1", "t2", "t3")))

ecl_long <- d2 %>%
 select(subject_id, page_flow, distraction, cueing, ECL_t1, ECL_t2, ECL_t3) %>%
 pivot_longer(cols = c(ECL_t1, ECL_t2, ECL_t3),
 names_to = "time_point",
 values_to = "ECL_score") %>%
 mutate(time_point = factor(time_point,
 levels = c("ECL_t1", "ECL_t2", "ECL_t3"),
 labels = c("t1", "t2", "t3")))

# TEST PHASE (t1 vs t3)
icl_test <- icl_long %>% filter(time_point %in% c("t1", "t3"))
ecl_test <- ecl_long %>% filter(time_point %in% c("t1", "t3"))

# TRAINING PHASE (t2 only)
icl_training <- icl_long %>% filter(time_point == "t2")
ecl_training <- ecl_long %>% filter(time_point == "t2")

# RM-ANOVA: TEST PHASE (t1 vs t3)

anova_icl_test <- aov(ICL_score ~ time_point * cueing * distraction +
 Error(subject_id/time_point),
 data = icl_test)
anova_ecl_test <- aov(ECL_score ~ time_point * cueing * distraction +
 Error(subject_id/time_point),
 data = ecl_test)

cat("\n--- ICL TEST PHASE RM-ANOVA ---\n")

##
## --- ICL TEST PHASE RM-ANOVA ---

print(summary(anova_icl_test))

##
## Error: subject_id
## Df Sum Sq Mean Sq
## cueing 1 1.85 1.85
##
## Error: subject_id:time_point
## Df Sum Sq Mean Sq
## time_point 1 2.383 2.383
##
## Error: Within
## Df Sum Sq Mean Sq F value Pr(>F)
## time_point 1 0.21 0.2129 0.316 0.575
## cueing 1 0.23 0.2254 0.334 0.564
## distraction 1 1.19 1.1876 1.761 0.186
## time_point:cueing 1 0.15 0.1484 0.220 0.639
## time_point:distraction 1 1.33 1.3318 1.975 0.161
## cueing:distraction 1 0.05 0.0549 0.081 0.776
## time_point:cueing:distraction 1 0.28 0.2831 0.420 0.518
## Residuals 224 151.06 0.6744

cat("\n--- ECL TEST PHASE RM-ANOVA ---\n")

##
## --- ECL TEST PHASE RM-ANOVA ---

print(summary(anova_ecl_test))

##
## Error: subject_id
## Df Sum Sq Mean Sq
## cueing 1 3.467 3.467
##
## Error: subject_id:time_point
## Df Sum Sq Mean Sq
## time_point 1 0.3475 0.3475
##
## Error: Within
## Df Sum Sq Mean Sq F value Pr(>F)
## time_point 1 0.2 0.1745 0.113 0.737
## cueing 1 0.2 0.1591 0.103 0.749
## distraction 1 0.5 0.5347 0.345 0.557
## time_point:cueing 1 0.5 0.4870 0.314 0.576
## time_point:distraction 1 0.1 0.0815 0.053 0.819
## cueing:distraction 1 0.0 0.0179 0.012 0.914
## time_point:cueing:distraction 1 0.0 0.0260 0.017 0.897
## Residuals 224 346.9 1.5485

# Between-subjects ANOVA: TRAINING PHASE (t2)

aov_t2_icl <- aov(ICL_score ~ cueing * distraction, data = icl_training)
aov_t2_ecl <- aov(ECL_score ~ cueing * distraction, data = ecl_training)

cat("\n--- ICL TRAINING PHASE ANOVA (t2) ---\n")

##
## --- ICL TRAINING PHASE ANOVA (t2) ---

print(summary(aov_t2_icl))

## Df Sum Sq Mean Sq F value Pr(>F)
## cueing 1 0.93 0.9306 1.779 0.185
## distraction 1 0.10 0.0968 0.185 0.668
## cueing:distraction 1 0.06 0.0639 0.122 0.727
## Residuals 113 59.12 0.5232

cat("\n--- ECL TRAINING PHASE ANOVA (t2) ---\n")

##
## --- ECL TRAINING PHASE ANOVA (t2) ---

print(summary(aov_t2_ecl))

## Df Sum Sq Mean Sq F value Pr(>F)
## cueing 1 8.04 8.044 9.163 0.00306 **
## distraction 1 1.39 1.387 1.580 0.21141
## cueing:distraction 1 0.35 0.345 0.393 0.53184
## Residuals 113 99.20 0.878
## ---
## Signif. codes: 0 '***' 0.001 '**' 0.01 '*' 0.05 '.' 0.1 ' ' 1

### PLOTS
# 1. ICL Line Plot with Error Bars
p_icl_line <- ggplot(icl_summary, aes(x = time_point, y = mean_icl,
 color = cueing, group = cueing)) +
 geom_line(size = 1.2, position = position_dodge(0.1)) +
 geom_point(size = 3, position = position_dodge(0.1)) +
 geom_errorbar(aes(ymin = mean_icl - se_icl,
 ymax = mean_icl + se_icl),
 width = 0.1, position = position_dodge(0.1)) +
 facet_wrap(~ factor(distraction, levels = c("noDistractions", "distractions")),
 labeller = label_both) +
 scale_x_discrete(labels = c("T1", "T2", "T3")) +
 scale_color_brewer(type = "qual", palette = "Set1") +
 labs(title = "Intrinsic Cognitive Load (ICL) Across Time Points",
 subtitle = "Error bars represent standard error",
 x = "Time Point",
 y = "Mean ICL Score",
 color = "Cueing Condition") +
 theme_bw() +
 theme(axis.text.x = element_text(angle = 0, hjust = 0.5),
 plot.title = element_text(hjust = 0.5),
 plot.subtitle = element_text(hjust = 0.5))

# 2. ECL Line Plot with Error Bars
p_ecl_line <- ggplot(ecl_summary, aes(x = time_point, y = mean_ecl,
 color = cueing, group = cueing)) +
 geom_line(size = 1.2, position = position_dodge(0.1)) +
 geom_point(size = 3, position = position_dodge(0.1)) +
 geom_errorbar(aes(ymin = mean_ecl - se_ecl,
 ymax = mean_ecl + se_ecl),
 width = 0.1, position = position_dodge(0.1)) +
 facet_wrap(~ factor(distraction, levels = c("noDistractions", "distractions")),
 labeller = label_both) +
 scale_x_discrete(labels = c("T1", "T2", "T3")) +
 scale_color_brewer(type = "qual", palette = "Set1") +
 labs(title = "Extraneous Cognitive Load (ECL) Across Time Points",
 subtitle = "Error bars represent standard error",
 x = "Time Point",
 y = "Mean ECL Score",
 color = "Cueing Condition") +
 theme_bw() +
 theme(axis.text.x = element_text(angle = 0, hjust = 0.5),
 plot.title = element_text(hjust = 0.5),
 plot.subtitle = element_text(hjust = 0.5))

# 5. Individual Trajectories for ICL (Spaghetti Plot)
p_icl_spaghetti <- ggplot(icl_long, aes(x = time_point, y = ICL_score, group = subject_id)) +
 geom_line(aes(color = cueing), alpha = 0.4) +
 geom_smooth(aes(group = cueing, color = cueing),
 method = "lm", se = TRUE, size = 1.5, alpha = 0.8) +
 facet_wrap(~ distraction, labeller = label_both) +
 scale_x_discrete(labels = c("T1", "T2", "T3")) +
 scale_color_brewer(type = "qual", palette = "Set1") +
 labs(title = "Individual ICL Trajectories Across Time Points",
 subtitle = "Individual lines and group trends colored by cueing condition",
 x = "Time Point",
 y = "ICL Score",
 color = "Cueing Condition") +
 theme_bw() +
 theme(plot.title = element_text(hjust = 0.5),
 plot.subtitle = element_text(hjust = 0.5))

# 6. Individual Trajectories for ECL (Spaghetti Plot)
p_ecl_spaghetti <- ggplot(ecl_long, aes(x = time_point, y = ECL_score, group = subject_id)) +
 geom_line(aes(color = cueing), alpha = 0.4) +
 geom_smooth(aes(group = cueing, color = cueing),
 method = "lm", se = TRUE, size = 1.5, alpha = 0.8) +
 facet_wrap(~ distraction, labeller = label_both) +
 scale_x_discrete(labels = c("T1", "T2", "T3")) +
 scale_color_brewer(type = "qual", palette = "Set1") +
 labs(title = "Individual ECL Trajectories Across Time Points",
 subtitle = "Individual lines and group trends colored by cueing condition",
 x = "Time Point",
 y = "ECL Score",
 color = "Cueing Condition") +
 theme_bw() +
 theme(plot.title = element_text(hjust = 0.5),
 plot.subtitle = element_text(hjust = 0.5))

# Display all plots
print(p_icl_line)


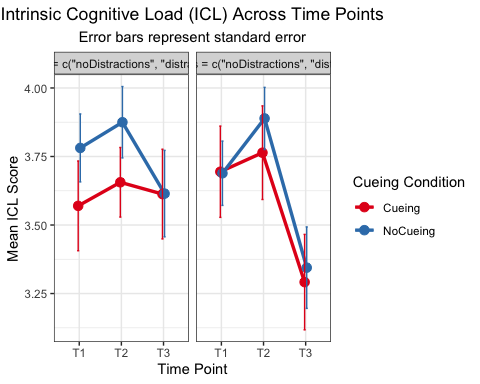


print(p_ecl_line)


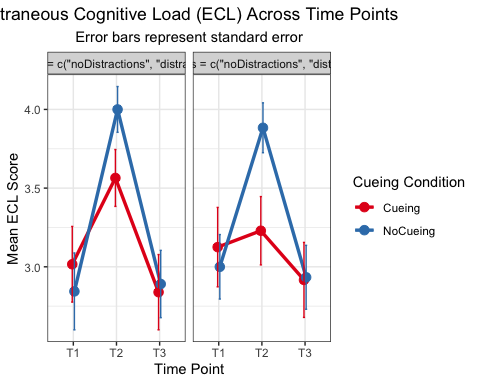


print(p_icl_spaghetti)

## `geom_smooth()` using formula = 'y ~ x'


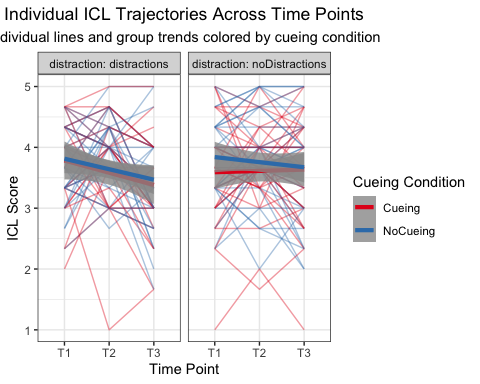


print(p_ecl_spaghetti)

## `geom_smooth()` using formula = 'y ~ x'


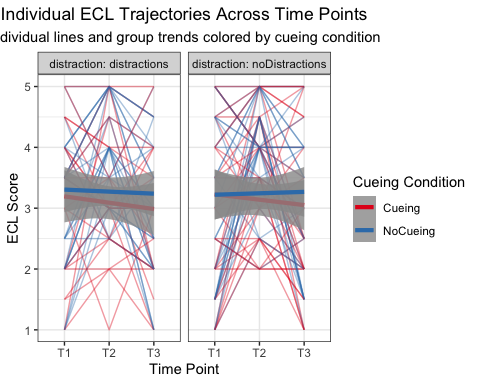


# Print summary statistics
print("ICL Summary Statistics by Condition and Time Point:")

## [1] "ICL Summary Statistics by Condition and Time Point:"

print(icl_summary)

## # A tibble: 12 × 7
## time_point cueing distraction mean_icl sd_icl se_icl n
## <fct> <fct> <fct> <dbl> <dbl> <dbl> <int>
## 1 ICL_t1 Cueing distractions 3.69 0.816 0.167 24
## 2 ICL_t1 Cueing noDistractions 3.57 0.912 0.164 31
## 3 ICL_t1 NoCueing distractions 3.69 0.643 0.117 30
## 4 ICL_t1 NoCueing noDistractions 3.78 0.701 0.124 32
## 5 ICL_t2 Cueing distractions 3.76 0.837 0.171 24
## 6 ICL_t2 Cueing noDistractions 3.66 0.707 0.127 31
## 7 ICL_t2 NoCueing distractions 3.89 0.621 0.113 30
## 8 ICL_t2 NoCueing noDistractions 3.88 0.737 0.130 32
## 9 ICL_t3 Cueing distractions 3.29 0.853 0.174 24
## 10 ICL_t3 Cueing noDistractions 3.61 0.911 0.164 31
## 11 ICL_t3 NoCueing distractions 3.34 0.814 0.149 30
## 12 ICL_t3 NoCueing noDistractions 3.61 0.892 0.158 32

print("ECL Summary Statistics by Condition and Time Point:")

## [1] "ECL Summary Statistics by Condition and Time Point:"

print(ecl_summary)

## # A tibble: 12 × 7
## time_point cueing distraction mean_ecl sd_ecl se_ecl n
## <fct> <fct> <fct> <dbl> <dbl> <dbl> <int>
## 1 ECL_t1 Cueing distractions 3.12 1.24 0.252 24
## 2 ECL_t1 Cueing noDistractions 3.02 1.34 0.240 31
## 3 ECL_t1 NoCueing distractions 3 1.12 0.205 30
## 4 ECL_t1 NoCueing noDistractions 2.84 1.38 0.244 32
## 5 ECL_t2 Cueing distractions 3.23 1.06 0.217 24
## 6 ECL_t2 Cueing noDistractions 3.56 1.01 0.181 31
## 7 ECL_t2 NoCueing distractions 3.88 0.868 0.158 30
## 8 ECL_t2 NoCueing noDistractions 4 0.823 0.145 32
## 9 ECL_t3 Cueing distractions 2.92 1.17 0.238 24
## 10 ECL_t3 Cueing noDistractions 2.84 1.33 0.239 31
## 11 ECL_t3 NoCueing distractions 2.93 1.11 0.203 30
## 12 ECL_t3 NoCueing noDistractions 2.89 1.21 0.214 32

###### ADDITIONAL DESCRIPTIVES
desc_stats <- d_change %>%
 select(age, gender, ultrasound_prkno, preparation, SUM_ANATOMY_PERFORMANCE)

print("Descriptive Statistics:")

## [1] "Descriptive Statistics:"

print(summary(desc_stats))

## age gender ultrasound_prkno preparation
## Min. :19.00 male :42 Min. :1.000 Min. :1.000
## 1st Qu.:20.00 female:75 1st Qu.:1.333 1st Qu.:1.000
## Median :20.00 Median :1.667 Median :2.000
## Mean :21.09 Mean :1.775 Mean :2.017
## 3rd Qu.:21.00 3rd Qu.:2.333 3rd Qu.:3.000
## Max. :40.00 Max. :3.333 Max. :4.000
## SUM_ANATOMY_PERFORMANCE
## Min. :1.000
## 1st Qu.:3.000
## Median :4.000
## Mean :4.684
## 3rd Qu.:6.000
## Max. :9.000

print("Extended Descriptive Statistics (numeric variables):")

## [1] "Extended Descriptive Statistics (numeric variables):"

print(describe(select(desc_stats, -gender)))

## vars n mean sd median trimmed mad min max range
## age 1 117 21.09 2.35 20.00 20.65 1.48 19 40.00 21.00
## ultrasound_prkno 2 117 1.77 0.59 1.67 1.73 0.49 1 3.33 2.33
## preparation 3 117 2.02 1.03 2.00 1.91 1.48 1 4.00 3.00
## SUM_ANATOMY_PERFORMANCE 4 117 4.68 1.97 4.00 4.59 1.48 1 9.00 8.00
## skew kurtosis se
## age 4.89 33.88 0.22
## ultrasound_prkno 0.56 -0.40 0.05
## preparation 0.66 -0.77 0.10
## SUM_ANATOMY_PERFORMANCE 0.41 -0.44 0.18

print("Gender Distribution:")

## [1] "Gender Distribution:"

print(table(desc_stats$gender))

##
## male female
## 42 75
